# Lower-status experts' influence on health-care managers' decision-making

**DOI:** 10.1108/JHOM-11-2020-0446

**Published:** 2021-08-27

**Authors:** Roy Liff, Ewa Wikström

**Affiliations:** Gothenburg Research Institute , Gothenburg, Sweden; Department of Business Administration, School of Business, Economics and Law at University of Gothenburg , Gothenburg, Sweden

**Keywords:** Health care, Identity positioning, Lower-status experts, Process alignment, Strategic alignment

## Abstract

**Purpose:**

The purpose of this paper is to investigate and theoretically explain how line managers and lower-status experts work together in public health-care organizations. Hence, this study explores how lower-status experts influence line managers' decision-making and task prioritizing in order to guide staff experts' cooperation and performance improvements.

**Design/methodology/approach:**

The authors used a qualitative method for data collection and analysis of the experts' and line managers' explanations about their cooperation. A theoretical approach of experts' identity positioning, in terms of differences and similarities, was used in analyzing the interaction between managers and experts.

**Findings:**

This study shows that similarities and differences in positioning acts exist simultaneously. Similarity is constructed by way of strategic and professional alignment with the line managers' core tasks. Differences stem from the distinction between knowledge-grounded skills and professional attributes such as language, analytical tools, and jargon. Lower-status experts need to leave their entrenched positions and match the professional status of line managers in both knowledge aspirations and appearance to reach a respected approach of experts' identity positioning.

**Originality/value:**

Unlike many previous studies, this study demonstrates that similarities and differences in positioning acts exist simultaneously.

## Introduction

In public organizations, there has been an expansion of support functions in staff positions due to new public management ( Ackroyd *et al.* , 2007 ). In health-care organizations, human resource (HR) experts and other quality experts in staff positions have been added to the organization ( Wadmann *et al.* , 2019 ). Including experts is supposed to strengthen the line managers' ability to improve the performance of public organizations ( Andrews *et al.* , 2009 ; Rosenberg Hansen and Ferlie, 2016 ). Relationships between line managers and experts in staff positions constitute a pertinent area of study regarding the structure of public organizations ( Weber, 1946 ; Dalton, 1950 ; Mintzberg, 1979 ; Sapir *et al.* , 2016 ). Experts in staff positions have extensive knowledge or abilities in specific areas, based on research, experience or occupation ( Styhre *et al.* , 2010 , pp. 159–160). Both experts and line managers may have extensive knowledge and be professionals. However, not all experts may be professionals. Professionals have exclusive rights to perform work associated with their specific area of scientifically-grounded theory, acknowledged in professional licensing and legislation (Abbot, 1988). Different professionals hold different statuses, depending on their authority in relation to other professionals ( Freidson, 2001 ). New professionals may hold relatively lower status than established ones, such as physicians ( Abbott, 1988 ; Freidson, 2001 ). Experts may differ from line managers, who may well have extensive knowledge, and be professionals. Experts in staff positions constitute part of the external environment of line managers working in the primary operations. Given that this staff position exists alongside positions in the line organization, we assume that staff experts lack formal authority to exert power over the line managers' actions and decision-making ( Crozier and Friedberg, 1980 ).

Researchers ( McGuire *et al.* , 2008 ; Thilander, 2013 ) have indicated that the main problem with experts is their inability to influence the line managers' operational and strategic decision-making, whereas the managers' main difficulty is underdeveloped control over or collaboration with the experts. Line managers consider the staff experts' knowledge to be inadequate and interfere in their attempt to achieve their core goals ( Sapir *et al.* , 2016 ; DiBenigno, 2019 ). Line managers do not value the experts' knowledge and believe that it is harmful to follow their recommendations ( Huising, 2015 ; Valentine, 2018 ).

We claim that a consequence of the increased number of experts in health care organizations is increased organizational complexity for managers in governing, as long as experts and line managers have difficulties cooperating.

Previous research on cooperation difficulties have addressed the need for experts to connect with line managers' organizational knowledge ( Freidson, 1970 ; Truss, 2009 ; Arena *et al.* , 2010 ) to make their problem-solving methods visible and to demonstrate their unique competence in a relationship with the managers' problem definitions ( Dutton *et al.* , 2001 ; Mikes, 2009 ; Hall *et al.* , 2015 ). In these studies, and others concerning cooperation between experts and mangers, similarity in knowledge or perspective between cooperating actors concerning the experts' alignment with management's processes and strategies is presented as a success factor ( Ashford *et al.* , 1998 ; Dutton *et al.* , 2001 ; Howard-Grenville, 2007 ). An explanation for the importance of the experts' alignment in previous studies may be that the experts have recognized knowledge and skill to exercise their expertise, but one that is difficult for the managers to understand. Experts have difficulty exerting influence because staff experts play peripheral roles, lack the opportunity to offer advice and lack the relational foundation that line experts rely upon to identify strategic management questions ( DiBenigno, 2019 ). Peripheral experts face difficulties gaining respect as equal to the managers and are not regarded as strategic assets ( Sandholz *et al.* , 2019 , p. 1,350). According to DiBenigno (2019 , p. 29), peripheral experts who take advantage of windows of opportunity early in the relationship exert influence over line manager's decision-making in a “rapid relationality.”

The recipients of the advice present additional difficulties with staff experts in their attempts to exert influence; line managers may regard themselves as having extensive knowledge based on scientifically grounded theories that are superior to the staff experts' pretensions on expertise. However, if the manager depends on the advice ( Kellogg, 2019 , pp. 950–951), if the advice supersedes the managers' knowledge, or if it is central to the workflow ( Black *et al.* , 2004 , pp. 600–601), then the manager regards the expert's knowledge as adequate. These studies highlight the need for experts to identify and provide their indispensable knowledge and skills to the managers' core tasks. Experts are considered as belonging to lower-status occupational groups than the line managers, who regard the experts as representing a lower level of professional status than the line managers see themselves as occupying ( Black *et al.* , 2004 ). They consider the staff experts as peripheral, lower-status experts.

There have been studies on the barriers faced by experts, such as lack of formal authority ( Crozier and Friedberg, 1980 ); lack of relevant knowledge of the core processes ( Arena *et al.* , 2010 ) and, given the boundaries between staff and line, lack of a relational foundation ( DiBenigno, 2019 ). However, there is still a need to examine how peripheral experts with lower professional status exert influence over the decisions made by line managers with higher professional status, as in the relation between physicians and staff experts in health care organizations.

The aim of this study is to investigate and to provide a theoretical explanation on how line managers and lower-status experts work together in public health care organizations. Hence, we explore how lower-status experts influence line managers' decision-making, and task prioritizing, in order to guide staff experts' cooperation and performance improvements. This is of special interest since many new categories of experts have been added to public organizations, including health-care organizations, with high expectations that they will improve these organizations; at the same time, we know from previous studies that this is difficult for experts in general. It may be assumed that these difficulties could be pronounced for experts with a lower professional status than that of the line managers. This study aims to contribute to a theoretical understanding of how such lower status experts act and can gain influence.

Through interviews, we followed the efforts of occupational health service (OHS) experts such as company physicians, nurses, physiotherapists/ergonomists, behavioral/organizational consultants, health pedagogues and engineers in an occupational health unit as they attempted to influence the line managers' decision-making in health care organizations in matters affecting occupational healthcare. All these OHS experts may be regarded as peripheral to their staff positions, and, except for the company physicians, may be regarded as lower-professional-status experts in relation to the line-manager physicians in this study. This assumption is in line with Freidson's (2007) theory on professional dominance in healthcare, whereby physicians enjoy the highest prestige of all professions.

How the OHS experts in this study influenced line managers in providing rehabilitation services is a reference point for the degree to which the experts were able to exert influence in the services they provided from a lower-status position. The study's setting offered the opportunity to investigate the impact of experts in a lower-status position, regardless of other possible influencing conditions.

A theoretical approach of expert identity positioning, in terms of similarities and differences, enabled us to analyze the interaction between managers and experts. The experts in our study struggled to become more influential vis-à-vis the managers' decision-making by mobilizing positioning acts related to how they simultaneously upheld *both* differences and similarities in their interactions with the managers. Therefore, we contribute to the literature by (1) identifying the experts' patterns of interactions with line managers associated with positioning acts related to the line managers' operative decision-making and (2) theorizing about experts' positioning acts in interaction with line managers and their patterns of similarities and differences.

## Previous explanations for how experts become influential

Although few studies have specifically addressed how peripheral, lower-status, occupational experts become influential, there are studies on technicians and physicians (e.g. Black *et al.* , 2004 ), technical writers and engineers ( Sapir *et al.* , 2016 ), and HR strategists, and line managers ( Sandholz *et al.* , 2019 ). According to Black *et al.* (2004) , lower-status (their computed tomography (CT)-scan technicians were called “lower-power experts”) may influence line managers (their physicians were called “higher-power experts”) if they have superior knowledge or technical skills that higher-power experts depend on for their operations. As Black *et al.* (2004) demonstrated, however, if these physicians believed technicians were dominating the technology, they withdrew from interactions with technicians in the CT-scanning activity. The hierarchical position of the physicians' diagnostic authority superseding the technician's technological authority was upheld although the boundary between the two categories was blurred when the technicians acquired some diagnostic competence and the physicians gained some technological competence. However, when the physicians gained more knowledge of CT technology than the technicians, physicians relegated the technicians to a minor role and the traditional pattern of professional dominance occurred. Black *et al.* 's overall conclusion was to recognize that “a balance of expertise across occupational boundaries in operating the technology creates a pattern in which the benefits of the new technology are likely to be realized most rapidly” (p. 572).

Sapir *et al.* (2016) studied the interaction of peripheral and core experts in creating new cross-occupational knowledge through such dialogical mechanisms as learning, inquiry, negotiation and knowledge sharing. They concluded that a fruitful interaction mechanism created a bond between the actors, allowing for “tolerance and mutual attentiveness on the part of the participants” (p. 33), creating new knowledge through mutual respect. However, Sandholz *et al.* (2019) showed that it is difficult for professionals (HR experts in their study) to redefine their profession to higher-status work through what Sandholz *et al.* call jurisdictional entrenchment. They claimed that acknowledged professional jurisdiction by the surrounding actors' results has positive and negative consequences: It serves as a protection of the professional domain and an obstacle to its revitalization. Pate *et al.* (2010 , pp. 208–211) argue that for lower-status professionals, there is even a counteracting mechanism between achieving professional identification and interprofessional partnership. Comeau-Vallée and Langley (2019 , p. 1,668) express a nuanced view in explaining lack of success in interprofessional relations' lower-power professionals, meaning they will have difficulties in reaching consensus within its group, which in turn increases the difficulties in gaining interprofessional recognition.

## A contextual framework

How experts exert influence has been studied previously. Dutton *et al.* (2001) , for example, demonstrated the process whereby various experts influenced the strategic agenda of top management through “issue selling” – moves that include *packaging, involvement* and *timing* . *Packaging* comprises alignment of the message to the company's business plan and presentation of facts in the correct order and form; *involvement* requires the coordination of similar targets of interest to the right people; and *timing* involves waiting for the right moment to raise an agenda item (p. 732). Dutton *et al.* emphasized that managers with an area of expertise can become influential only if they “move” (process) based on an understanding of the thought patterns of innovations (strategy and managerial skill).

Accordingly, the process in the interaction between experts and top management matters, but the strategic dimension is also central if it serves as an “internal engine” for change and innovation in the strategic decision-making of top management. Dutton *et al.* (2001) suggested that successful issue sellers draw on relational, normative and strategic contextual knowledge, using various moves to achieve their objectives, including a characterization of their appeal as a rational incremental step tied to key organizational goals and priorities. Early in the process, they involve a wide range of colleagues, particularly people at or above their level. They keep their bosses informed and persist in their selling efforts, while taking advantage of timing to help them decide when to sell their ideas and when to hold back.

Hall *et al.* 's (2015) study also elaborated on issue selling, developing an understanding of situated interactions between experts and managers. They showed that the central role of toolmaking is “structural arrangements and interpersonal connections when explaining how functional experts can become influential” (p. 3), arguing that accuracy of information is not critical for exerting influence, but that “communicability” and “experts' personal involvement” are essential (p. 19). Thus, experts become influential through formal procedures such as meetings and informal procedures such as trust in interpersonal relationships. According to Hall *et al.* (2015) and similar to Dutton *et al.* (2001) , the first condition is the process by which toolmaking is used in order to align the experts' information or area of expertise in relation to the managers' operations: “the process by which experts adopt, adjust and reconfigure tools that embody their (and others') expertise – plays a vital role” in explaining how experts may become influential in relation to managers ( Hall *et al.* , 2015 , pp. 18–19). The second condition is strategic and is described as how experts become influential in relation to managers' strategic agenda, by aligning their area of expertise/information to that of the managers in their strategic decisions, “where experts may (or may not) find the ‘privileged moments' to interact with business managers, whether in compliance roles, discretionary strategic decisions, or in performance evaluation and planning” ( Hall *et al.* , 2015 , p. 19).

Pritchard (2010) observed that earlier efforts to examine experts' attempts at becoming strategic have usually taken a role perspective. According to Pritchard (2010 , p. 185), the experts' pursuit of influence could be regarded as an active performance of identity work rather than a changing role description or new tasks framed as a new job specification. She suggests that focusing on expert identity positioning in the conversations and interactions of everyday work activities should guide the research on experts' ability to gain influence. Since this approach concerning the experts' professional identity seems especially adequate in a study concerning lower-status experts, we followed her recommendation.

## Positioning and concepts used in exploring experts' positioning acts

Czarniawska (2013) suggested a theoretical approach for positioning acts, which could serve as a useful frame for analyzing organizational events in terms of social dynamics. This approach highlights an interactive production of selves, comprising ongoing positioning acts – in organizing practices with various outcomes, for example, in which experts can simultaneously stress their *similarities* and *differences* in managerial concerns and problems in achieving recognition and influence ( Czarniawska, 2013 ). The concepts of similarity and difference are fundamental to the dialectic of positioning acts. As Jenkins (2008) asserted, “Taken, as they can only be – together – similarity and difference are the dynamic principles of identification, and are at the heart of the human world” (p. 15). This process has been attributed to self-positioning, defined by Sveningsson and Alvesson (2003) as “forming, repairing, maintaining, strengthening or revising the constructions [of self] that are productive of a sense of coherence and distinctiveness” (p. 1,165). It can be assumed there is an inherent tension in striving for both similarities and differences ( Lewis, 2000 ; Jenkins, 2008 ).

In the study of OHS experts' positioning acts and in agreement with Kreiner *et al.* (2006) , we have assumed that people's search for a balance between similarities (“belonging,” in the Kreiner *et al.* 's terminology) and differences (“uniqueness” for Kreiner *et al.* ) must be negotiated within a social structure in which we include practice and place – plus discourse. Similarities and differences must be negotiated in the social structures of work groups and departments at various managerial levels, and in their external relationships.

We combined Dutton *et al.* 's (2001) concept of issue selling with Hall *et al.* 's (2015) concept of common construction of tools. Both concepts concern different ways of constructing similarities. We identified two categories of similarities for our analysis: *strategic alignment,* including *packaging;* and *process alignment,* with its two sub-categories: *communicativeness/meaning-creation* and *common toolmaking* .

1. *Strategic alignment* refers to experts' involvement in an organization's strategic business plan and alignment of the message in relation thereto – experts' participation in specific situations or specific issues of interest to managers, for example.
2. *Process alignment* refers to experts' personal involvement in an experts' approach in convincing managers to use their methods when the managers try to understand the context and conditions of the relevant strategic activities, allowing the experts to exhibit their competence and demonstrate their actions and achievements. This approach includes common toolmaking, in which timing is a key element.

Based on Freidson's (1970 , 2001) work, we identified two categories of *differences* : *use of technical skills* and *professional attributes* .

1. *Use of technical skills* refers to the adoption of theoretically based models and conceptualizations of knowledge in practice (e.g. evidence-based methods).
2. *Professional attributes* refer to the use of professional, institutionalized jargon and external factors (e.g. study rooms, dress codes and such exercises of authority as medical responsibility).

Thus, starting with Czarniawska's (2013) idea that identity builds on both similarities and differences, we have found in the existing literature what has been regarded as important similarities in the relationship between line managers. These relate to the use of strategic alignment, including packaging and process alignment with its two sub-categories: communicativeness/meaning creation and common toolmaking to create similarities (see Dutton *et al.* , 2001 ; Hall *et al.* , 2015 ). Similarly, we found in the existing literature (See Freidson, 1970 , 2001 ) that the *use of technical skills and professional attributes* creates differences between experts and line managers. We examine how the *use of technical skills* and *professional attributes* creates differences (See Freidson, 1970 , 2001 .)

Because similarities and difference are continuous rather than discrete variables, we examined the degree of each. We also examined the extent to which the actors achieved a balance in their similarities and differences using attributive positioning and self-positioning ( Czarniawska, 2013 ). Czarniawska concluded that negotiations about the “self” arise mainly from two types of positioning acts: attributive positioning (in this case, how managers acknowledge experts' position) and self-positioning (how the experts position themselves). The two key outcomes of an individual's positioning activities are acceptance or rejection of the self-positioning.

## Occupational health services: the study's setting

The occupational health unit is an internal organization within a large regional organization (RVG (Region Västra Götaland)) in Sweden, mainly comprising several hospitals and health centers, to which the occupational health unit should provide services. Hospital managers, recipients of the OHS experts' advice, are traditionally physicians, with their own high pretensions on expertise. The occupational health unit has a reputation of success in exerting influence over managerial decision-making in rehabilitation, but not in the other two services provided – work-related illness prevention and promotion of employee well-being – and their superiors had asked the experts why they were unsuccessful in all their services. Perhaps the differences in how the respondents value the service are due to different legal regulations. The rehabilitation service is supported by legal responsibilities imposed on managers and by the defining procedures the managers must follow. The state's regulatory role has obviously arranged for the rehabilitation experts to achieve influence over the line managers' decision-making. That is not the case in the areas of work-related illness prevention and employee well-being, leaving the OHS experts providing these services with lower-status positions than what the line managers enjoy.

Our research deals with the work of OHS experts employed in a 90-person section of the county council's 50,000-employee organization. The OHS organization is composed of company physicians, nurses, physiotherapists/ergonomists, behavioral/organizational consultants, health pedagogues and engineers. Client organizations have engaged OHS experts mainly within statutory ( AFS, 1994: 1 ) rehabilitative areas, approximately 80% in individual cases. The main task of company physicians and nurses is to engage in this service.

The latest Swedish work environment legislation ( AFS, 2015: 4 ) has increased the focus on prevention and health promotion, making these services the responsibility of employers and managers. These services constitute the primary tasks of physiotherapists/ergonomists, behavioral/organizational consultants, health pedagogues and engineers although rarely with more than one service in the same organization. OHS experts must be involved in the operational plans and practices of client organizations although agreements often lack clear cooperation goals or conditions and methods for creating health-promoting workplaces. Contracts are established annually, based on estimated service required that year, particularly in rehabilitation, health promotion and contracts negotiated case by case. Client organizations have their own HR managers, each of whom works with a line manager in the client organization, in cooperation with the OHS experts.

The client organization's HR managers usually function as the point of contact to the OHS experts, as HR managers interact with union parties and line managers. HR managers assist line managers in recruitment and systematic work environment issues, and often support line managers in their OHS activities, seeking the role of strategic advisors to managers at all levels.

The setting of this study is a hospital – similar to the archetypical acute care institution ( Glouberman and Mintzberg, 2001a , b ) in which central coordinating mechanisms are usually embedded in the hierarchy, in managing the coordination between managers and experts as “the standardization of outputs” and “the standardization of work.” However, as Glouberman and Mintzberg described, in situations where outputs and work cannot be standardized, it requires other types of coordination between managers and experts based on mutual judgment. This situation exists *in hospitals* when experts, such as occupational health experts, are positioned in a peripheral staff unit (support function outside of the unit's operations) to support line managers inside the organization in solving the unit's complex, everyday issues. The line managers are organized in a two-tier system: on the operational level are the unit managers, often nurses, and on the upper level are the department managers, often physicians. Overall, physicians dominate the decision-making concerning major issues, which, for example, could emanate from the OHS experts' suggestions. Glouberman and Mintzberg describe this type of coordination as “flexible communication among peers, so that the unexpected can be dealt with adaptively and collaboratively” (p. 75). They emphasize the role of mutual adjustments, in which managers and experts respond to each other's ideas – in solving complex administrative problems, for example. This means that the hospital setting provides a fertile ground for studying how line managers with high a professional status interact with experts having a lower professional status *.*

## Research methodology

### Design and data collection

We used a qualitative method for data collection and analysis of the experts' and line managers' explanations about their cooperation. We used the three main service activities of rehabilitation, prevention and promotion to describe and analyze experts' attempts to gain influence, focusing on process alignment, strategy alignment, and the distinctions of technical skills and professional attributes. It may be assumed that experts who together perform a service become integrated into a team and have their primary loyalty to the team and the clients for whom they work ( Cregård, 2018 ). Thus, we assume that an organizational position as cooperating experts is more compelling for the lower-status experts with low professional status than their professional background. We described and analyzed rehabilitation as the reference service to the other two services and defined their levels of achievement in relation to the rehabilitations service, regarded as the ideal type of case. This is because the OHS physicians are mostly involved in rehabilitation service,s and as experts, they enjoy the highest level of prestige of the OHS experts. Moreover, they are also involved in the OHS service that has the longest and most formal power position in relation to the line managers.

We interviewed OHS experts and line managers of RVG's client organizations – organizations responsible for their employees' work environment. We interviewed HR managers – staff employees within the client organizations – who often assist line managers, both department and unit managers, in their interactions with external OHS experts. These line manager–HR manager dyads were regarded as one unit by the OHS experts.

We initially met with the OHS management team to obtain data for guiding subsequent individual interviews. We conducted 40 individual interviews:

1. Twenty-three interviews with managers, which included 11 interviews with first line managers (nine nurses, three behavioral/administrative background) and 12 interviews with HR managers.
2. Seventeen interviews with OHS experts [company physicians (5), company nurses (2), physiotherapists/ergonomists (5), behavioral/organizational consultants (2), health pedagogues (2), and engineers (1)].

We aimed for variety in size and service type among the RVG organizations that volunteered for participation. Individuals were interviewed face-to-face and alone; we asked them to reflect on their experience of interaction with each other, beginning with open-ended questions, followed by more specific questions intended to encourage rich, detailed answers and reduce general and theoretical digressions. The interviews revealed how the OHS experts and line managers describe and understand their activities and how they perceive their cooperation. The responses guided us in finding examples of situations in which first-line managers had experienced difficulties and successes in cooperation with the OHS experts. The interviews were audio-taped and transcribed.

### Analysis and data presentation

We analyzed the interview data, with particular reference to participants' descriptions of their efforts to collaborate in the rehabilitation, prevention and health-promotion services. This analysis required three repeated rounds of coding, as we examined the purpose, actions and achievements of these three service activities.

In Round 1, we reviewed our empirical data account-by-account, noting our first-impression reflections. During the coding, we labeled the accounts in the interview transcripts, using patterns and connections in their descriptive content ( Kvale, 2006 ; Corbin and Strauss, 2008 ). We were particularly interested in whether the data indicated that the experts used a positioning approach, which could be interpreted as a process by which they tried to produce an identity and seek other actors' acceptance of that identity.

In Round 2, we used the theoretical framework derived from the literature for the categorization process and developed a selection of theoretical categories ( Charmaz, 2006 ; Denzin and Lincoln, 2011 ). It concerned positioning acts and introduced concepts to our analysis, with its identification of similarities and differences as our analytical guide. We used the concepts of *strategic alignment* and *process alignment* (sub-categories of *communicativeness/meaning-creation* and *common toolmaking* ) to analyze similarities and *technical skills* and *professional attributes* (sub-categories of *professional jargon* and *external factors* ) to analyze differences. In Round 2, we matched interview comments to these categories in a second review.

In Round 3 of our analysis, we referred to Czarniawska's (2013) discussion of attributive positioning and self-positioning. We examined the data in each category from Round 2 regarding the experts' positioning activities as they tried to become strategic resource partners with the line managers, focusing on the three service activities: rehabilitation, prevention and promotion.

In Round 4, we made some observations of similarities and differences across professional groups among experts to ascertain how, if at all, the professional identity of the OHS experts influences their strategic priorities.

See Table 1 for the theoretical categories and examples of categorization for rehabilitation, prevention and health promotion.

### Credibility of the study

The credibility of the study depends on the authenticity of the data, that is, the respondents in their answers reflect what they actually experienced (lived experience) and not what they think or consider as appropriate to answer to make the interviewer happy or to present their business or themselves ( Yin, 2003 ). In this study, this was avoided as much as possible by the respondents reproducing concrete examples of experiences from OHS. Based on these, they evaluated how the experts and line managers have acted in the interaction with their counterpart and in this way given them the opportunity to recollect lived experience ( Van Maanen, 1997 ). We attended several meetings to demonstrate our commitment and to gain insight into the problems and the agendas facing the respondents. In order to build commitment and to get feedback from managers and OHS experts, we attended and presented the findings at three different types of meetings: (1) five meetings with the management group at the OHS, (2) three reflection meetings (one with all involved line managers, one with all HR managers, and one with all OHS experts), and (3) three focus group interviews comprising two groups of both biomedical and behavioristic staff (a total of 11 individuals), and one group of OHS managers at a first line level (five individuals). By attending meetings and following a procedure, we were able to verify the research findings; moreover, the feedback also supported the validity of the findings. Furthermore, as Czarniawska (2007) has suggested, the ability to blend into the organization increases the probability of obtaining authentic answers.

### Ethical considerations

Before we conducted the interview study, we informed the respondents about the purpose of the study. They were also informed that participation was voluntary and could be ended at any time. By providing a written consent, they agreed to participate. Personal contact data for the respondents were only used to facilitate the scheduling of interviews.

The empirical material was anonymized, and the names of the respondents have not been identified in order to avoid ethical problems, given the possible sensitive nature of commenting upon the experts' skills and ways of acting. The personal contact data as well as the empirical material will be stored at the university in a safety cabinet for ten years. The respondents were informed about the right to access their personal contact data, in accordance with the Data Protection Regulation.

## The three main services and attempts to gain influence

### Employee rehabilitation – the reference service

Line managers – usually nurses – in the client organization's employee rehabilitation often initiate contact directly. A nurse or psychologist from OHS with established social positions in employee rehabilitation prepares rehabilitation proposals from a work ability evaluation, aimed at effective employee rehabilitation and sick leave reduction. The process is clearly described: (1) managers initiate contact directly with HR to resolve issues related to labor legislation and co-workers' proper work with rehabilitation activities and (2) managers initiate contact directly with OHS experts – issues related to arranging meetings with co-workers and co-workers' proper work with rehabilitation activities (see more examples in Table 1 ).

I initiate contact with a nurse or physician at the occupational health services when an employee in my unit has been in hospital for a long time. First, I try to work on behalf of the employee for a while, but if it seems like a long sick leave, I contact my HR partner and the Occupational Health Services. (First-Line Manager)

And as an OHS nurse said, “All cases of employee rehabilitation are handled by the OHS experts, even if we, in turn, sometimes use external experts on specific treatment methods.”

*Process alignment* is achieved largely through standardized cooperation among HR, the unit manager, insurance providers and OHS experts, with individual responsibilities specified step by step. OHS's contracts with client organizations align professional expertise with managers' problems by putting action in its context.

The rehabilitation examples show that OHS experts create *strategic alignment* through packaging, in relation to the managers' legislatively mandated responsibility to prepare employees' rehabilitation plans. The managers' interactions with OHS experts and the client organization's HR manager regarding employee rehabilitation and work ability evaluation meet the managers' contractual responsibilities. OHS experts and the HR manager then enter strategic alignment in the rehabilitation work, consistent with the managers' formal assignments. OHS experts use their own terminology, which defines rehabilitation as a professional activity comprising specific talk therapy techniques, physiological tests, forms and protocols to achieve the *distinction of technical skills* – a bridge between self-positioning and available theoretical models, and a key condition in the construction of social positions in strategic alignment with other actors ( Freidson, 1970 , 2001 ).

The rehabilitation plan reflects the *distinction of professional attributes* , their specific *professional jargon* and the work ability evaluation by professionals with exclusive authority for these tasks. The results of the study show that the attributive positioning of rehabilitation is high, both in similarities and differences. The OHS experts accept and adjust to attributed positioning. The line managers and HR managers who request OHS experts expect the OHS experts to cooperate with rehabilitation legislation. OHS experts' actions to establish themselves as strategic resources and partners with the rehabilitation practice managers focus on the use of common medical knowledge and interaction with HR plans. This practice has preventive benefits; standardizations of accounts and procedures, with evidence-based practice; and advances in education qualifications. The similarities and differences in the rehabilitation services are all well developed.

It is doctors, psychologists and nurses who are mainly the three professions that perform this service. They show a high degree of congruence in their actions toward the client organization, both in terms of similarities, as they jointly follow institutionalized forms for both processes and strategic alignment. Psychologists, to a somewhat lesser degree than doctors, have the opportunity to make a distinction regarding technical skills and professional attributes. However, in this role with their science-based methods, they can largely achieve the same degree of difference as doctors. The nurses have an assisting role to primarily the doctors and also their own professional attributes, some of which are congruent with those of the doctors.

### Prevention of work-related illness

In this category of service, we find examples of such concrete activities as “ergonomic adaptation of the workplace” (OHS expert) and the mandatory biennial medical examinations of all employees (fitness tests, blood samples and discussion of health habits), for which managers buy health profile analyses from the OHS experts. Employees with borderline unhealthy habits or employees who fall within the risk zone for illness may converse with a health coach regarding physical and psychosocial factors. As one first-line manager stated, “We conduct health analyses and health profiles, together with OHS. We look at employees' stress and well-being, primarily highlighting medical condition: blood pressure and fat content in the blood, for example.” These analyses are recorded for follow-up.

For this service, the actors use an HR expert, an extended medical, and a financial paradigm: workplace ergonomic adjustments, fruit baskets, and work-break walks; discussions on the negative aspects of certain lifestyle choices; and financial logic to support the advantages of a healthy employee group. This extended paradigm requires the interaction of line managers, HR managers and OHS experts. There are still difficulties, however, in detecting and processing early, weak, signals of upcoming problems. An OHS psychologist explained,

In order to capture the signals, it is necessary to have some kind of clear process flow regarding how they should be identified, and the process should be clear to HR and line management and OHS agreement. It also contains various elements and specific, concrete parts. And I don't think we have this in our team. We don't have an agreement with receivers, but this we will do. It doesn't come from the receiver that is [the] HR [manager] or from the line managers who say: “Okay! Now we're curious. What you have found. This we need to work with.” Without that, it is something that we perceive as a mission, but we haven't really agreed that this is what we should do. And then, there's not really a receiver. (OHS psychologist).

*Process alignment* is achieved when the health profile analyses are included in the annual contract, and the budget is set in agreement with HR managers from the client organization or with head-office managers. The statement from the psychologist indicates, however, that the process alignment is insufficient when the prevention measurements concern organizational aspects.

Thus, OHS experts enter *strategic alignment* through packaging when managers' responsibility for the work environment, including employees' preventative activities, is delegated to OHS experts. The OHS uses specific terminology to define prevention as a professional activity. For example, OHS experts express their unique competence by using such terminology as “ergonomic review and adjustment,” “health profiles,” “lecture on lifestyle and leadership,” and “methods for conflict resolution.” OHS experts in preventive healthcare connect their self-positionings (descriptions of unique competences, responsibilities and positionings of various actors) to theoretical models concerning prevention (extended medical theoretical models and theoretical models concerning individual employees, prevention and the work environment) (See e.g. Watson, 2008 ; Freidson, 1970 , 2001 .). OHS experts also use the *distinction of technical skills* through test cycles and protocols, medical diagnosis techniques, health analyses and profiles, ergonomic treatments, lifestyle lectures and such structured therapies as talk therapies. They also use the *distinction of professional attributes* when employees meet OHS's white-coated physicians and nurses in a medical-professional environment.

The results of our study indicate that the attributive positioning of rehabilitation is stronger than that of preventative measures. Freelance experts, for example, can provide nutrition and exercise advice. In its self-positioning, OHS experts seem to have little interest in increasing the differences between themselves and these external freelancers; rather, they seek greater alignment with process and strategy. The outcome of the positioning act of prevention work is also accepted, albeit on a lower level of similarities and differences than rehabilitation work. The similarities and differences in prevention services are summarized as *strategic alignment* is somewhat developed, p *rocess alignment* is well developed, *differences constructed through technical skills* is well developed, and *professional attributes through professional* jargon is somewhat developed.

It is physiotherapists/ergonomists, behavioral/organizational consultants and engineers who mainly perform this service, but doctors and nurses are also involved. Similarities in the three professions' actions can be maintained for the sub services that are regulated in the annual agreement and which are conducted as package services under terms such as “ergonomic review and adjustment.” Sub-services related to organizational aspects can be led by either of the mentioned professions and can then be considered to be characterized by the leading OHS expert's professional affiliation, which in that case can reduce the congruence in the professionals' implementation. The same may apply for the different professionals to demonstrate differences in a common way. For organization-related initiatives, difficulties in achieving difference are indicated in general through technical skills and professional attributes, regardless of which profession leads such assignments.

### Promotion of employees' well-being

This study reveals few concrete examples of work and interaction in health promotion, which one first-line manager described as an “on-going dialogue with managers and co-workers on how to improve the workplace environment.” HR shows the work organization aspect by cooperating with OHS experts, linking their competences and expert knowledge with managers regarding the issues raised in employee questionnaires:

One example is the work with the employee survey. We may need to start with a dialogue, although not necessarily with someone from OHS. I want all managers to feel that they have an HR consultant with whom to discuss their responses. We also need the opportunity to turn to OHS for complex situations. Then, we need other skills. So, we offered soup lunches, where managers could meet to discuss employee surveys. (HR manager)

Health promotion may include organizational structure and transforming of work practices, in which strategic decisions and structures deal with the organization's responsibilities: patient care, organizational logic aimed at sustainable work practices, and creation of healthy work organizations. The medical secretaries' project (reduction of sick leave and improved working conditions) is one example we learned about in which OHS experts were seen as a resource in a project with consequences for organizational structure. One HR manager suggested reorganization, from a central department to the original structure, in which medical secretaries were linked to the wards directly and organizationally.

Two preparatory meetings were held before we arrived. At the first, HR managers discussed the project's purpose and ways of arranging the change process. Ward managers were invited to the second meeting. In six months, medical secretaries were to be invited to join project groups to analyze their workplace problems and discuss solutions. When asked how OHS experts were to be engaged, the HR manager answered: “I think it's a good idea to involve OHS [experts] earlier. They offer different input. Now they come in late, where it may be too late to save the situation or resolve the conflict.”

This study revealed examples of work environment issues in relation to large change processes that may begin with new building plans, changes in practices or alternative management models. We conclude that OHS experts have difficulty entering an organization using organizational or psychological approaches with managers. Their contribution seems to be perceived as a complication for managers, who tend not to address problems as they arise, requiring OHS experts to be called into the organization, anyway.

*Process alignment* in health promotion is not achieved through agreed-upon procedures or routines, as in rehabilitation, nor through accepted commodification and contracts that set prices, as in the prevention of work-related sick leave. Nor is *strategic alignment* achieved with the managers. Using OHS experts to resolve organizational issues seems sporadic and fragmented – that OHS experts are “discovered” as a potential resource in strategic or operational decision-making in the absence of process alignment or strategic alignment. However, managers' discussions on employee surveys and informal events such as soup lunches may be understood as a desire to create process alignment and strategic alignment through action-in-context.

From a positioning perspective in health promotion, OHS experts have no well-defined positionings, and there are diffuse discourses concerning health promotion that could serve as interpretations of the difficulties in creating a process and strategic alignment among professionals from the various organizations. OHS experts cannot create clear bridges between available theoretical models (on organization, health promotion and sustainable organizational practices) and self-positionings (clear descriptions of their unique competences, responsibilities and positionings) (See e.g. Watson, 2008 ; Freidson, 1970 , 2001 ).

OHS experts lack specific terminology and *distinction of technical skills,* defining health promotion as a professional activity. OHS experts use few terminologies to describe health promotion services: “salutogenic” (as opposed to pathogenic – health supporting rather than disease-causing factors) and what an OHS expert described as “educating line managers in health promotion leadership.” Some line managers in the customer organizations particularly enjoy working with health promotion, and highlighted areas that OHS experts could explore: strategic decisions, organizational structures and organizational assignment, for example. Neither managers nor OHS experts and HR managers have professional attributes to be used in communications about health-promotion activities. OHS experts lack key conditions – a distinction between technical skills and professional attributes – in constructing social positionings ( Watson, 2008 ).

This study highlighted that attributive positioning of health-promotion work is not accepted because it lacks both similarities and differences. Line managers, HR experts and OHS experts are unable to connect to health-promotion work. OHS experts who want to be better aligned with line managers sometimes take the initiative together with HR managers to arrange soup lunches with the line managers We found no descriptions of initiatives that increase the differences in health-promotion work, however. In contrast to rehabilitation and prevention, analyses of attributive positioning and self-positioning of health-promotion work show few similarities and differences. The overall outcome of this positioning act is that other relevant core actors are denied positioning. The similarities and differences in the health promotion services are summarized as following: *strategic alignment* is lacking (except sporadically), *process alignment* is lacking (except sporadically), *differences constructed through technical skills* is weakly developed or missing and *professional attributes through professional jargon* is weakly developed or missing *.*

It is behavioral/organizational consultants and health pedagogues who mainly perform this service. Regardless of which of these professions is most influential in performing a task within this service, they lack the necessary alignment to achieve similarity and also what is required to demonstrate difference. Both of these professions see it as their task within this service to educate line managers in health promotion leadership with the same approach of trying to be invited to management teams to talk about health promotion. Potential differences between the experts in this approach, however, do not show up in the study.

In summary, our results reveal that OHS experts' efforts to become strategic resources and partners with managers involve positioning acts with different combinations of similarities (process alignment and strategy alignment) and differences (distinction of technical skills and professional attributes). Moreover, when cooperation is established with managers, the different combinations of similarities and differences have consequences for the outcome of the positioning acts. A successful cooperation includes many similarities *and* differences, and positioning is successful when experts and managers accept the positioning acts. The analysis in Round 4 confirms that the position in a team is more decisive for the experts acting than their professional background.

## Discussion

This article has addressed “How lower-status experts manage to influence line managers' decision-making?”

We argue that experts' interactions with frontline managers in rehabilitation work are an expression of strategic alignment achieved through compatible goal sharing and striving for mutual benefit, as previously observed ( Dutton and Ashford, 1993 ; Mohr and Spekman, 1994 ). Our study also confirms these conclusions. These findings exhibit what Caldwell (2003) included in his definition of the strategic partner role, in which the expert becomes influential in the managers' strategic decision-making and agenda. OHS experts have established the necessary relationship with frontline managers in rehabilitation work and have framed their practices as elements of the managers' organizational management, which Pritchard (2010) considers necessary prerequisites to a successful cooperation.

Prior studies have often concluded that experts' differences separate them from other organizational actors and have suggested a search for similarities in which the packaging, timing, toolmaking ( Dutton *et al.* , 2001 ; Howard-Grenville, 2007 ; Hall *et al.* , 2015 ), or language-as-management tools in communication ( Styhre, 2002 ) explain the experts' success in exerting influence outside of their specialized areas.

Previous research on lower-status experts have urged these experts to gain respected positioning as equals to core professionals ( Sandholz *et al.* , 2019 , p. 1,350), either by providing solutions to problems crucial for the operations of core actors ( Black *et al.* , 2004 ) or by exerting a skillful dialogical interaction with core actors ( Sapir *et al.* , 2016 ). This study confirms the importance of lower-status experts being recognized as influential and as equal to the core actor.

The contribution of this study is that it illustrates a way for lower-status experts to achieve respected positioning: to manifest a high degree of similarity – and especially a high degree of difference. Difference is reflected in professionalism – demonstrating technical skills based on legitimate knowledge and the accepted attributes of their specific professions, just like the core actors do in their work. To be regarded as important, one must go from being an expert to being an influential professional in line managers' decision-making – just like the core actors do. It seems necessary to undergo a transition of the knowledge base and a transition in communicability in order to overcome jurisdictional entrenchment ( Sandholz *et al.* , 2019 ) and be regarded as strategic by core actors.

## Conclusion

The aim of this study was to investigate and to provide a theoretical explanation on how line managers and lower-status experts work together in public health-care organizations, which was achieved by exploring how lower-status experts influence line managers' decision-making, and task prioritizing, in order to guide staff experts' cooperation and performance improvements. This study contributes to an understanding of how experts exert influence over managers' operational decision-making by simultaneously upholding differences and similarities with line managers. Previous research concerning the interaction between experts and managers usually emphasizes the necessity of similarities. But experts in the unsuccessful examples in this study appear to have exhibited too little difference. It appears that the necessary similarities are not achieved by avoiding differences. This is reflected by using positioning acts as a theoretical framework – which has not been used previously in research on workplace safety or in an investigation into experts' actions toward increasing their influence on line managers' decision-making. With this evidence from a public-sector organization, we add to previous studies within the general research field of experts' influence on managers ( Dutton *et al.* , 2001 ; Howard-Grenville, 2007 ; Hall *et al.* , 2015 ).

We argue that using this framework and the evidence regarding interactions between experts and managers may aid in understanding how lower-status experts exert influence over the decision-making of managers. Peripheral, lower-status, experts must create legitimacy for their expertise by bridging their self-positioning and available theoretical models (See e.g. Watson, 2008 ; Freidson, 1970 , 2001 ). With a lack of connection between self-positioning and the available theoretical model, experts cannot construct social positioning acts as grounds for making their knowledge relevant to managers and influencing decision-making. Black *et al.* (2004 , pp. 600–601), Kellogg (2019 , pp. 950–951) and DiBenigno (2019) all recommend an adaptation to preconditions; in accordance with Sandholz *et al.* 's (2019) research, our study emphasizes the need for managers to acknowledge experts' unique contributions through their knowledge base and regard them as equals. Moreover, it emphasizes the need for managers to understand their dependence on expert recommendations in achieving their organization's core goals.

*Practical implications:* Our study also shows that experts who link their practice and recommendations to scientific knowledge can gain an influence over managers' decision-making, if they can prove that the scientific base of their competence and skills has a status and legitimacy equal to that of the managers' knowledge and skills. Lower-status experts need to match the professional status of line managers also in appearance, by using professional attributes to reach a respected approach for experts' identity positioning.

*Future research:* It would be appealing to study a case that allowed us to describe in greater detail how identity is performed in the actual work of the unit analyzed – a study that requires observational data, preferably collected longitudinally. A way to study the experts' strategies in greater detail would be to investigate whether the experts employ different strategies depending on what category the line managers belong to and differences between how different kinds of line managers, for example, if they are doctors or nurses, perceive the strategies of experts providing the same category of occupational health service. However, the varying responses from the different managers indicate that it would be difficult for them to differentiate between the strategies with experts delivering the same service. We have made some preliminary observations of similarities and differences across professional groups among experts involved in the three different services. However, this may also be studied more thoroughly in future studies. It would also be intriguing to investigate the question we studied here in an expanded context: the effect on the decision-making line managers who are subject to influence by the internal experts, external experts and semi-external experts like the OHS experts interviewed in this study. We assume that the results are, to a large extent, even if not strictly, transferable to other hospitals and the relationship between line managers and various types of experts in hospitals, *also outside of Sweden,* given that the client organizations we studied are like the archetypical acute care institutions described by Glouberman and Mintzberg (2001a , b) , and also given that OHS experts have a peripheral position similar to several kinds of other support functions held by different types of semi-professional experts that have emerged through the new public management ( Ackroyd *et al.* , 2007 ). This assumption is based on the argument presented by Alvesson and Robertsson (2016) that *“in-depth studies potentially offer new theoretical insights, the relevance of which is largely reliant on other researchers to reflect upon the subject matter and/or conduct further studies which add to our repertoire of understandings” (p. 14).* However, we would like to see this study extended to hospitals in other countries and to other industries. Even if our results prove to be valid for health care organizations outside of Sweden, in which the managers are physicians, they may not necessarily be generalizable to organizations with managers having a lower academic background – especially managers grounded in natural, empirical science with high demands for evidence-based statements. A similar study in a traditional bureaucracy inhabited by managers working as administrators and caseworkers would also be of interest.

Besides studies in other contexts, it would be of interest to enlarge or replace the theoretical framework used here to consider the importance of the degree to which the experts' concepts and tools have formalized knowledge to enhance management techniques ( Hatchuel and Weil, 1995 ).

## Figures and Tables

**Table 1:** Presentation of results and empirical examples on how OHS experts' identity work influences managers

	Identity work: similarities		Identity work: differences		Empirical examples
Services	Strategic alignment	Process alignment	Use of technical skills referring to theoretically based models	Professional attributes	
Rehabilitation	OHS experts contribute to managers' goal achievement and to fulfilling their responsibilities requested in rehabilitation legislation for co-workers	Common formalized processes involving managers, company physicians, nurses and HR specialists	Rehabilitation plan, especially use of diagnostic skills	Work evaluation Medical terminology dress code	Managers initiate contact directly with HR: issues related to labor legislation and proper work with rehabilitation activities by co-workers
					Managers initiate contact directly with OHS experts: issues related to arranging meetings with co-workers and proper conduct of rehabilitation activities by co-workers
Prevention	OHS experts influence managers' responses to the work environment and their formal work responsibilities with prevention	Process adaptation among managers, HR personnel and health-care experts' education	Ergonomic review and adjustment, health profiles and lectures on lifestyles. Vaccinations. Conflict resolution (by psychologists)	Co-worker surveys for opinions on work environment (ergonomics and health care workers)	Ergonomic adaptation of the workplace. Fruit baskets and exercise during rest periods. Wine boxes and weekend routines
					Personal issues from a financial perspective: discussions on finances related to co-worker's health as cost-reduction measures
Health promotion	Few empirical examples; participation particularly in initiatives, projects and specific measures for health	Few empirical examples: education and soup lunches	Few empirical examples for specific concepts	Reference to salutogenesis	Managers' leadership in health promotion
					Ongoing dialog with managers and co-workers on ways to improve workplace environment
